# COMMUNITY CENTERED PERSPECTIVES ON ALZHEIMER’S DISEASE: A SYNTHESIS OF DATA FROM FOUR COMMUNITY ADVISORY BOARDS

**DOI:** 10.1093/geroni/igae098.3643

**Published:** 2024-12-31

**Authors:** Ivy Ahmed, Diane DeVincentis, Teresa Cronin, Jennifer Pettis

**Affiliations:** Eisai, Nutley, New Jersey, United States; Eisai, Nutley, New Jersey, United States; Eisai, Nutley, New Jersey, United States; Gerontological Society of America, Clifton Park, New York, United States

## Abstract

As the number of people with Alzheimer’s disease in the United States continues to grow, they and their caregivers face increasingly complex challenges in the health care ecosystem. To understand the impact of Alzheimer’s disease on communities, Eisai with participation of the Gerontological Society of America convened four Community Advisory Boards (CABs) inviting a variety of communities of interest across multiple disciplines that serve the community. These informative day-long meetings shed light on the local impact of this potentially devastating disease and allowed for meeting participants to share many creative solutions in place through grassroots dedication. The team identified four different communities based on their unique population and location: Dade County, Florida; Summit County, Ohio; Greater Atlanta, Georgia; Los Angeles, California. During the meetings, participants reflected on the myriad needs within their community; highlighting their most pressing unmet needs; and spotlighted successful programs, initiatives, and interactions that occur within the community to support their community members with AD and their caregivers. While each community’s needs were unique, several common themes emerged uniting these four communities illustrating shared issues and creative solutioning. Shared themes included the need for materials that address brain health to raise awareness of AD prevention and the importance of early detection, culturally tailored resources, and information delivered by trusted community champions in settings that leverage existing social outlets/meeting places. This poster will illustrate key findings from each individual meeting and highlight commonalities identified among them.

